# KITLG Copy Number Germline Variations in Schnauzer Breeds and Their Relevance in Digital Squamous Cell Carcinoma in Black Giant Schnauzers

**DOI:** 10.3390/vetsci10020147

**Published:** 2023-02-11

**Authors:** Heike Aupperle-Lellbach, Daniela Heidrich, Alexandra Kehl, David Conrad, Maria Brockmann, Katrin Törner, Christoph Beitzinger, Tobias Müller

**Affiliations:** 1 LABOKLIN GmbH & Co. KG, 97688 Bad Kissingen, Germany; 2 Institut für Bioinformatik, Universität Würzburg, 97070 Würzburg, Germany

**Keywords:** tumour, toe, miniature schnauzer, standard schnauzer, CNV, ddPCR, breed predisposition

## Abstract

**Simple Summary:**

It is well known that dark-coated dogs, especially schnauzers, have a predisposition for digital squamous cell carcinoma (dSCC). Molecular genetic studies by other researchers indicate a pathogenetic role of copy number variations (CNVs) of the KIT ligand (KITLG) gene. Our study aimed to use droplet digital PCR of blood samples to investigate whether the copy number has a predictive value for the disease in schnauzer breeds. We showed that most giant (GS), standard (SS), and miniature schnauzers (MS) had more than four and up to seven copies of this gene segment. Furthermore, the copy number in black GS with dSCC was significantly higher than in the black control GS (*p* = 0.02). CNV values > 5.8 indicate a significantly increased risk for dSCC, while a reduced risk can be assumed for GS with CNV < 4.7. CNV values between 4.7 and 5.8 appear as a grey area. In summary, including the germline mutation (KITLG CNV) in breeding decisions will be complex, but this diagnostic test may help to assess the individual risk for dSCC and to sensitise owners of black GS accordingly if the CNV value is high.

**Abstract:**

Copy number variations (CNVs) of the KITLG gene seem to be involved in the oncogenesis of digital squamous cell carcinoma (dSCC). The aims of this study were (1) to investigate KITLG CNV in giant (GS), standard (SS), and miniature (MS) schnauzers and (2) to compare KITLG CNV between black GS with and without dSCC. Blood samples from black GS (22 with and 17 without dSCC), black SS (18 with and 4 without dSSC; 5 unknown), and 50 MS (unknown dSSC status and coat colour) were analysed by digital droplet PCR. The results are that (1) most dogs had a copy number (CN) value > 4 (range 2.5–7.6) with no significant differences between GS, SS, and MS, and (2) the CN value in black GS with dSCC was significantly higher than in those without dSCC (*p* = 0.02). CN values > 5.8 indicate a significantly increased risk for dSCC, while CN values < 4.7 suggest a reduced risk for dSCC (grey area: 4.7–5.8). Diagnostic testing for KITLG CNV may sensitise owners to the individual risk of their black GS for dSCC. Further studies should investigate the relevance of KITLG CNV in SS and the protective effects in MS, who rarely suffer from dSCC.

## 1. Introduction

Diseases of the canine toe can be of non-neoplastic (43%), neoplastic (52%), or tumour-like (5%) origin [1]. Squamous cell carcinomas (SCC), malignant melanomas, soft tissue sarcomas, and mast cell tumours are the most common malignant tumours of the toe [1,2,3,4].

Squamous cell carcinomas of the toes originate in the stratum spinosum of the claws or the sole epidermis. Clinically, they may initially look like inflammation of the nail bed. There may be lameness, thickening of the toes, and ulceration of the digital tissue. The nail material often appears softened and frayed [5]. Most commonly, the forelimbs are affected, and the mean age of the canine patients is about 10 years [4,5,6]. The tumour grows locally invasive and destroys the bone as it progresses [5,7,8]. In contrast to dogs, human SCC of the nail develops very rarely [9].

Amputation of the toe is the treatment of choice in dogs with dSCC [10]. The histological grade of malignancy can be assigned by two different systems adapted from human squamous cell carcinomas [11,12] to canine digital squamous cell carcinomas [13]. The risk of local recurrence can be considered low when resected in healthy tissue. However, multiple subungual squamous cell carcinomas on different toes of one dog have been described in several cases [4,6,14]. Varying metastasis rates depending on the breed have been described in the literature [5,6]. There may be lymph node metastases [5] or systemic spread affecting various organs [15].

Dark coat colour is correlated to the development and aggressiveness of squamous cell carcinoma [1,2,3,4,5,6,13,16]. A predisposition for squamous cell carcinoma in standard schnauzers (SS) and giant schnauzers (GS) has been identified by various authors [1,4,5,6,17]. In the United States and Canada, the giant schnauzer is among the ten breeds most commonly affected by toe amputations [2]. A data collection of 79 dogs from Italy showed that schnauzers were the most common breed (31.6%) in the study population and have a poorer prognosis than dogs of other breeds [5]. A large study from Canada confirmed the strong predisposition for the development of dSCC in giant schnauzers (odds ratio (OR): 56.7), standard schnauzers (OR: 20.3), Gordon setters (OR: 18.3), black standard poodles (OR: 11.1), Kerry blue terriers (OR: 9.4), rottweilers (OR: 7.0), and several other large black dog breeds [6]. In a German cohort of digital neoplasms, predisposed breeds for SCC included the schnauzer (log OR = 2.61), Briard (log OR = 1.78), rottweiler (log OR = 1.54), poodle (log OR = 1.40), and dachshund (log OR = 1.30) when compared with mongrels [1]. Interestingly, the miniature schnauzer (MS) seems to be rarely affected by squamous cell carcinoma [1,17]. This suggests the presence of genetic factors for the development of dSCC in dogs. Moreover, there is one report of dSCC in three members of a giant schnauzer family [18].

Cutaneous squamous cell carcinomas in man, which are mainly induced by ultraviolet light, have a different and more complex mutational landscape, e.g., with NOTCH1, NOTCH2, TP53, and RAS mutations [19]. Non-digital SCC in dogs shows the same high molecular genetic variability as human squamous cell carcinomas [20]. Thus far, canine dSCC has not been investigated for somatic oncogenic mutations.

Tyrosine kinase receptors (TKR) comprise more than 60 molecules that play an essential role in the molecular pathways of cell survival and differentiation [21]. The KIT receptor (CD117) is a TKR involved in the processes of survival and proliferation of mast cells, melanocytes, epithelial cells, and others [21]. Mutations in the c-kit gene encoding the KIT receptor are well known in canine mast cell tumours [22], in human melanomas [23], as well as in human and canine gastrointestinal tumours [24,25].

The ligand for this KIT receptor is the stem cell factor (SCF), which is encoded by the c-KIT ligand (KITLG) gene. This ligand seems to play a role in canine mast cell tumours [26], melanogenesis, and hair colour intensity in dogs [27] as well as in human familial progressive hyper- and hypopigmentation (FPHH) [28]. Overexpression of KITLG seems to be important in promoting lymph node metastasis via the JAK/STAT pathway in mouse models and cell lines of human nasopharyngeal carcinoma [29]. Furthermore, growth and invasion in human colorectal cancer cell lines are correlated with the expression of KITLG [30]. However, in human non-small cell carcinomas, no correlations were found between KITLG gene copy number, KITLG mRNA expression levels, or KITLG immunopositivity [31].

Copy number variations (CNVs) are defined as genomic regions that vary in number by amplification or deletion of DNA sequences. They play an important role in the genetic diversity necessary for evolution but are also responsible for hereditary and somatic human diseases, such as cancer [32]. Small CNVs are often benign, but those larger than 250 kb are strongly associated with pathological findings such as developmental disorders and neoplasms [33]. The KITLG CNV spans only 6 kb (chr15: 29,821,450–29,832,950) and is located 152 kb upstream of the KIT ligand (KITLG) gene [27]. A variable copy number alters KITLG expression and subsequent pigment distribution throughout the coat colour in dogs and several other species [34].

A genome-wide association study (GWAS) on standard poodle DNA found an increased risk of developing digital SCC when an increased number of specific copies (>4 copies) was expressed at the KITLG locus [35]. Furthermore, in nine dogs with digital malignant melanoma, the number of copies at the KITLG gene locus varied between four and six. Four of these nine dogs had black coats [36]. These studies were based on germline mutations detected in blood samples.

The aims of this study were (1) to investigate KITLG CNV in miniature, standard, and giant schnauzers and (2) to compare the CNV of KITLG between black giant schnauzers with and without digital squamous cell carcinomas and to evaluate its possible predictive value in this breed.

## 2. Materials and Methods

### 2.1. Animals and Material

#### 2.1.1. Study 1

Blood samples from 39 giant, 27 standard, and 50 miniature schnauzers were selected from the archive material stored after routine diagnostic examinations at LABOKLIN GmbH & Co. KG, Bad Kissingen, Germany (2014–2021). Inclusion criteria were that breed, age, and sex of the dogs were indicated on the submission form.

Samples from giant and standard schnauzers were obtained from pre-operative blood samples from animals with digital masses before amputation as well as from dogs with geriatric blood screening. The group of giant schnauzers (*n* = 39) included 13 male, 8 neutered male, 11 female, and 7 neutered female dogs aged 5 to 14 years (median 10 years). The group of standard schnauzers (*n* = 27) included 7 male, 7 neutered male, 5 female, and 8 neutered female dogs at the age of 5 to 13 years (median 10 years).

Clinical data of the standard and giant schnauzers included in study 1 were collected via questionnaires sent to the owners (breeders) and/or by telephone calls to the veterinarians. It was recorded whether the respective dogs had already suffered from SCC of the digit and which coat colour (black or pepper and salt) it had: All standard schnauzers and giant schnauzers in studies 1 and 2 were black.

Samples from miniature schnauzers mainly came from breeding animals (young and not neutered) that were routinely screened for relevant genetic diseases. The group of miniature schnauzers (*n* = 50) included 24 male and 26 female dogs between 1 and 9 years of age (median 1 year). Information about clinical findings or coat colour was not available.

According to the terms and conditions of LABOKLIN and the decision of the government of Lower Franconia RUF-55.2.2-2532-1-86-5, no special permission has to be obtained from the animal owners or the animal welfare commission for examinations on residual samples that are not needed for any further diagnostics.

#### 2.1.2. Study 2

Two groups of black giant schnauzers were selected from the cohorts of study 1:

(1) Control group (*n* = 11): Blood samples from black giant schnauzers aged >10 years (10–14 years; median 11.5) that remained free of digital SCC until the end of the study were used. There were 4 male dogs, 2 neutered males, 4 females, and 1 neutered female. Six younger dogs without dSCC from study 1 were excluded;
(2) dSCC group: Blood samples from 22 black giant schnauzers aged 6–13 years (median 10) with squamous cell carcinoma of the digit were diagnosed by different pathology laboratories. This group included 7 males, 6 neutered males, 6 females, and 3 neutered female dogs.

Clinical questionnaires for the standard schnauzers identified 18 black standard schnauzers with dSCC and 4 without dSCC. No data about dSCC were available in five cases; thus, they had to be excluded. As the control group of SS was too small, these data were not useful for a valid statistical analysis. We therefore did not conduct another study with these data.

SCC of the digit is not expected in the young miniature schnauzers of cohort 1. Furthermore, clinical data and coat colour were not available. Thus, no further statistical analyses were performed.

### 2.2. Molecular Genetics

To determine the copy number of the relevant gene segments of KITLG, molecular genetic analyses were performed on blood samples as follows: Genomic DNA was isolated from EDTA blood with the MagNA Pure 96 system using DNA Tissue Lysis Buffer and viral NA Small RNA kit (Roche, Basel, Switzerland) according to the manufacturer’s instructions. Copy number quantification of the KITLG was performed by digital droplet PCR (ddPCR) using TaqMan^®^ probes and primers specific for the KITLG sequence and proto-oncogene 1 (ETS1) as reference gene based on the paper of Bannasch et al. (2021) and as performed previously [36,37]. Measurement was take in duplicate. The mean value was used for further analyses. The intra-assay correlation was 0.85. The copy number was determined using the DropletReader (Bio-Rad, Feldkirchen, Germany) and QuantaSoftware 1.7.4.0917 (Bio-Rad, Feldkirchen, Germany).

### 2.3. Statistics

All statistical analyses and visualisations were carried out with the statistical framework R version 4.2 [37]. To compare the mean copy number values of KITLG in the three schnauzer groups (giant, standard, and miniature), we used the Kruskal–Wallis rank sum test. To compare the mean copy number values of the KITLG between the two breeds (giant schnauzer and standard schnauzer) with dSCC, we used the Wilcoxon rank sum test. Multiple logistic regression was employed for binary classification. To test which variables should be included in the multiple regression model, the Bayesian information criterion (BIC) was used as a model test. To estimate the expected accuracy of new data, we performed leave-one-out cross-validation (LOOCV) as implemented in the R package “boot” version 1.3–28.1 [38]. Throughout the whole manuscript, the following significance levels were used: *p* < 0.05 (weakly significant), *p* < 0.01 (significant), and *p* < 0.001 (strongly significant).

## 3. Results

### 3.1. Study 1

**Figure 1:**
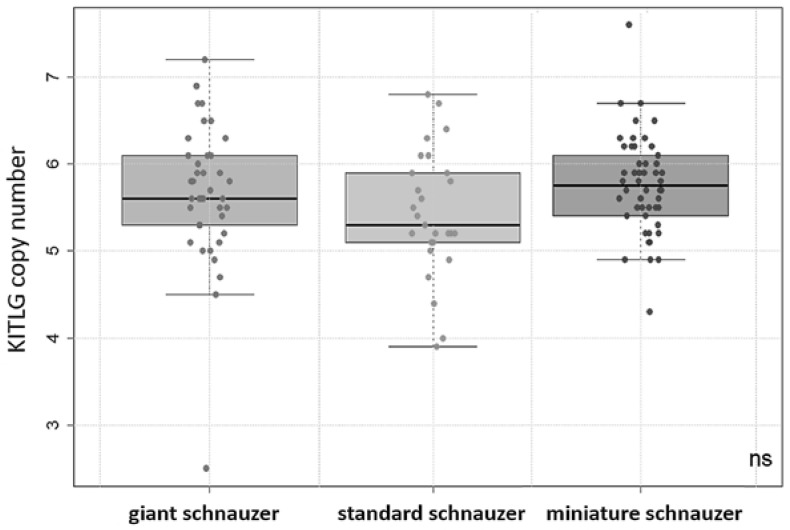
Boxplot: copy number values of KITLG in the giant (*n* = 39), standard (*n* = 27), and miniature (*n* = 50) schnauzer groups (including dogs with and without digital squamous cell carcinomas or unknown health status). There were no significant (ns) differences in the mean values between any of the breeds observed.

The copy numbers of KITLG in the cohort of study 1 (animals with and without dSCC) ranged from 2.5 to 7.6. There were no significant differences between the KITLG CNV values of the giant, standard, and miniature schnauzer groups tested by Kruskal–Wallis (Figure 1). Multivariate regression analysis showed that there was no significant effect of sex or castration status on the copy number value. Interestingly, there was one 13-year-old female giant schnauzer that had dSCC but had a very low copy number of 2.5. In this case, the histopathological diagnosis was confirmed in our laboratory, and analyses were repeated twice to prove this unexpected finding.

**Figure 2:**
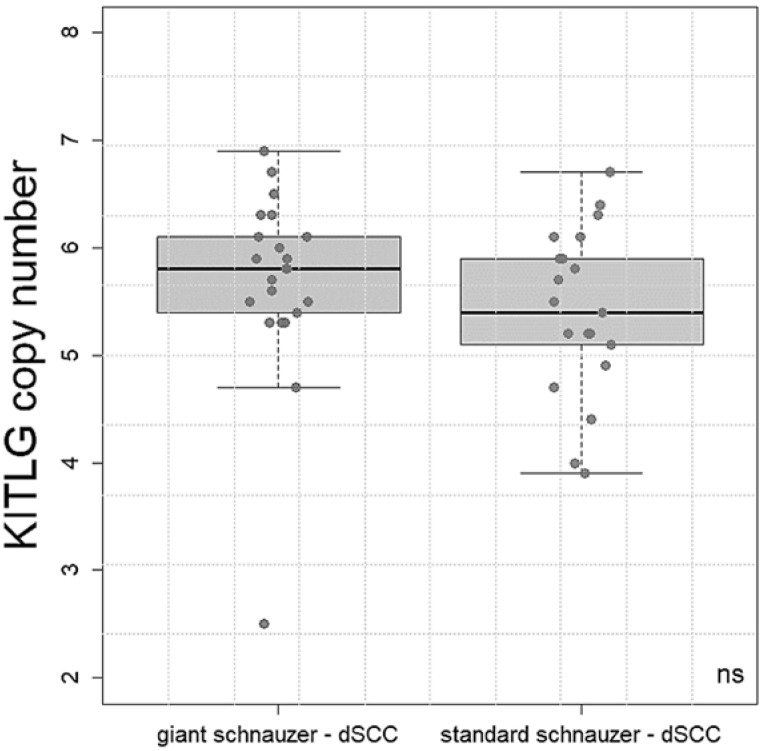
Boxplot: copy number values of KITLG in the giant (*n* = 22) and standard (*n* = 18) schnauzers with digital squamous cell carcinoma (dSCC). There was no significant (ns) difference in the mean values between the breeds.

The CN values form 18 standard schnauzers with dSCC ranged from 3.8 to 6.7 (median 5.4). The copy number values in 22 giant schnauzers with dSCC varied between 2.5 and 6.9 (median 5.7). There was no statistical significance between these two breeds (Figure 2).

### 3.2. Study 2

Of the 39 giant schnauzers of study 1, we selected 11 black giant schnauzers that had no SCC of the digit until the end of this study (controls) and 22 black giant schnauzers with digital SCC. In the dSCC group, 19 front legs and 3 hind legs were affected. Squamous cell carcinoma was diagnosed on all toes and was distributed as follows: toe I (*n* = 3), II (*n* = 7), III (*n* = 2), IV (*n* = 2), and V (*n* = 8). The other six GS without dSCC were younger than 10 years and were excluded from further statistical analyses because it was too uncertain whether they would develop dSCC later in life. Further medical history of neoplasia other than dSCC was available in only three dogs: One 13-year-old male GS never had any tumour. One 13-year-old female neutered GS died of cardiac insufficiency and suffered from diabetes insipidus. One 12-year-old female GS developed a non-specified mammary tumour after the end of this study.

**Figure 3:**
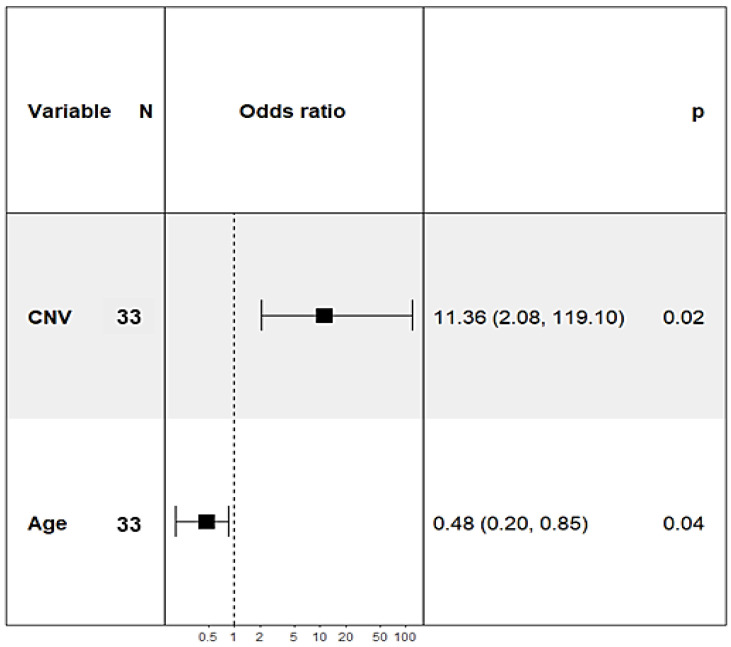
Forest plot: odds ratios of copy numbers of the KITLG locus and age varying significantly between the giant schnauzers with (*n* = 22) and without (*n* = 11) squamous cell carcinoma of the digit.

The number of KITLG gene copies in the control dogs ranged from 4.5 to 6.5 (median 5.2). The copy number in dogs with dSCC varied between 2.5 and 6.9 (median 5.7). The quality of the classification in giant schnauzers with and without SCC was analysed with a multiple logistic regression approach. It was identified that the copy number was significantly higher in the giant schnauzers of the dSCC group compared to the controls (*p* = 0.02, Figure 3). Furthermore, age was a weakly significant (*p* = 0.04) factor (Figure 2) and was adjusted in the statistical analyses. Sex did not have an impact, and there was no apparent tendency that the affected toe had any correlation with the copy numbers.

**Figure 4:**
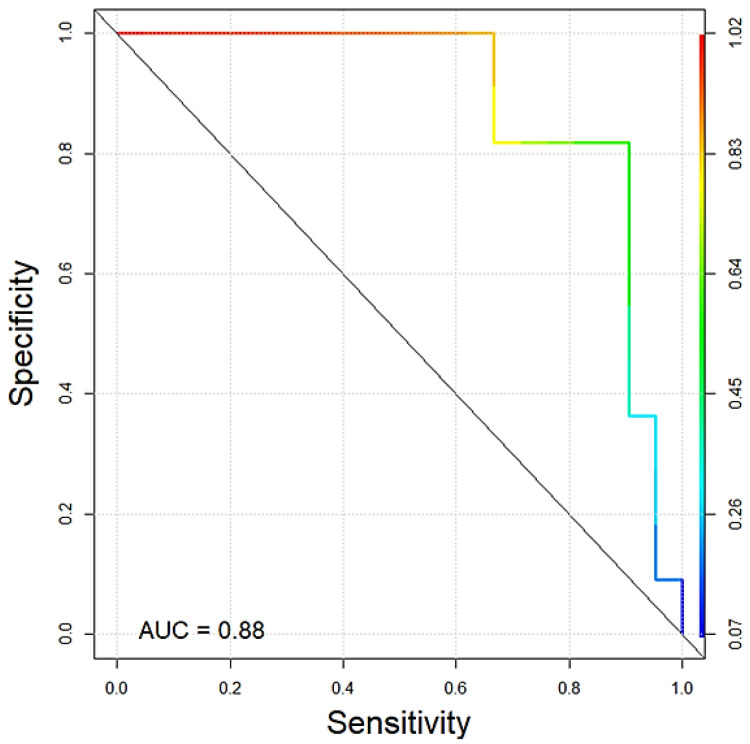
ROC curve showing the quality of copy number testing of the KITLG locus in black giant schnauzers with (*n* = 22) and without (*n* = 11) digital SSC of our cohort. The AUC value was 0.88.

An ROC curve was used to determine the specificity (true negative rate) and sensitivity (true positive rate) for predicting whether the sample of a giant schnauzer is classified as “control” or “dSCC”. In general, values of the area under the curve (AUC) can range from 0.5 (random noise) to 1 (optimal classification). The AUC value in the present analysis of copy number testing of the KITLG locus in black giant schnauzers was 0.88 (Figure 4).

**Figure 5:**
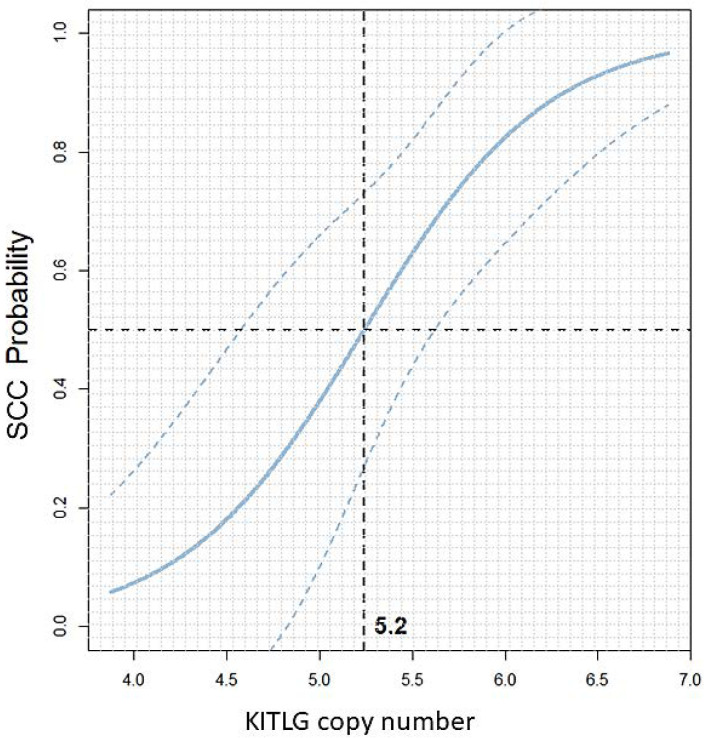
Scatterplot: KITLG copy number value versus the predicted dSCC probability in black giant schnauzers at the age of 10 years with 95% confidence intervals. The vertical dashed line at 5.2 indicates the 50% probability threshold. Giant schnauzers with a CNV value above this threshold have an increased probability of developing SCC of the digit, while CNV values below 5.2 indicate a lower probability of suffering from digital SCC. For an age-dependent analysis, please refer to Supplementary Figure S1.

To estimate the prediction quality on new samples, we applied leave-one-out cross-validation. This method yields an expected accuracy (percentage of correct predictions) of 81.25%. The results identified a 50% probability threshold of 5.2 copies with 95% confidence intervals for 10-year-old giant schnauzers as shown in Figure 5. Additionally, for the ages of 9, 11, and 12 years, the optimal thresholds for giant schnauzers are visualised in Supplementary Figure S1.

## 4. Discussion

Previous publications have identified that there is a basic risk of developing digital SCC in canine breeds with dark coats, such as standard and giant schnauzers [1,6,17]. In our study, all standard and giant schnauzers were black, and we could not investigate the effect of the coat colour variations that are common in schnauzer variants. We did not select black dogs with dSCC in our cohort, but at the time of collecting samples, all dSCC were from black MS and GS only. As described by other authors [4,5], the front legs were more often affected than the hind legs in the dogs in our cohort, and the median age was 10 years. A regular medical check-up of the toes of dark-coated dogs, especially black standard and giant schnauzers, should be recommended as a standard practice.

A KITLG copy number value >4 was found to be correlated with a predisposition to dSCC in black standard poodles compared with light-coloured standard poodles [35]. In a recent study, we found that 55 black-coated and black and tan dogs with dSCC had mean copy number values ranging from 5.5 to 5.8 in contrast to four light-coated animals with dSCC that had a mean copy number value of 4.5 [16]. Interestingly, this study has shown that there is a predictive correlation between higher germline KITLG copy number values and increased histological aggressiveness of digital SCC in dogs (mainly black-coated breeds and black and tan breeds) [16].

The present study was the first to compare the three size variants of the schnauzer breed for their genetic profile with regard to the copy numbers of the KITLG locus. Our data did not find any significant differences in CN values among the three breed variants we examined. The results of our study showed that all but one of the schnauzers had a copy number value >4 and are thus probably at-risk animals, as it has been defined for black poodles [35]. However, it is interesting to see in the literature that only standard and giant schnauzers are predisposed to develop dSCC, while miniature schnauzers are rarely affected [1]. Thus, there must be additional protective factors in miniature schnauzers that may be correlated to body size or coat colour but have not yet been identified. Future studies should evaluate the effects of coat colour in detail. Schnauzers were originally bred in Germany, and all the dogs of our study are kept in Germany, but there is no information on the pedigree and any genetic influence from schnauzers of other countries. According to the website of the German Pinscher and Schnauzer Klub (https://psk-projekt.jimdo.com/unsere-rassen, accessed on 5 February 2023), German giant and standard schnauzers are mainly black (puppies per year: 1 pepper and salt GS for every 10 black GS and about 1 pepper and salt SS for every 1.8 black SS). In contrast, the coat colour of miniature schnauzers varies between black, pepper and salt, black-silver, and white (18.04.2007/EN FCI-Standard N° 183). The coat colour of the puppies per year is: 1 white to 1.3 black-silver to 3.4 black to 4.7 pepper and salt (https://psk-projekt.jimdo.com/unsere-rassen, accessed on 5 February 2023).

As we only had black SS and GS in our study, we investigated whether there are any differences in KITLG copy numbers between black giant schnauzers with or without dSCC during their life. The number of standard schnauzers with reliable clinical information was too small for a valid statistical analysis.

Yet, for the first time, a predictive statement could be made about the probability of SSC occurring in giant schnauzers. We found that in black giant schnauzers, more copies of the KITLG locus were significantly associated with the probability of developing dSCC (*p* = 0.02). One limitation of our GS control group is that it is based on the assumption that these animals have not developed squamous cell carcinoma of the digit even when older than 10 years. However, we have designed this group according to the control group of the standard poodles (older than 8 years) in the initial study mentioned above [35].

For diagnostic purposes in black GS, we recommend defining values between 4.7 and 5.8 as a grey area. A copy number value of more than 5.8 indicates a significantly increased risk for digital SCC in black giant schnauzers. A reduced risk can be assumed for black giant schnauzers with a copy number below 4.7. Further studies with higher case numbers are needed to confirm the results of this pilot study.

Nevertheless, further factors are likely to contribute to the development of such tumours, as there was one old giant schnauzer (13 years) with dSCC that had a CNV of 2.5. In this case, spontaneous dSCC can be assumed, which is independent of factors of breed predisposition.

However, the copy numbers in black standard and giant schnauzers with dSCC did not vary significantly. As we were not able to collect enough samples from control standard schnauzers by the end of this study, further investigations are necessary to determine similar diagnostic thresholds for this breed. Furthermore, this phenomenon should be analysed comparing black and pepper and salt standard schnauzers as well.

In contrast, miniature schnauzers very rarely develop digital SCC [17] but also had copy numbers ranging from 4.3–7.6 in our cohort. Thus, at the moment, the results of a KITLG CNV test should be restricted to black giant schnauzers. For the future, it is important to understand the oncogenic mechanisms and the influence of body size and/or coat colour in the schnauzer breed variants in more detail.

Thus far, only very few studies have investigated the KITLG (stem cell factor) expression level on mRNA or protein levels in human cells [30], mouse models [29], or canine samples [26]. However, no correlation was found between KITLG gene copy number, KITLG mRNA expression levels, or KITLG immunopositivity in human non-small cell carcinomas [31]. Further studies should investigate this correlation in cases of digital squamous cell carcinomas in dogs. Presuming that the ligand of the KIT receptor plays a role in oncogenesis, one would have to assume that the receptor itself is expressed in squamous cell carcinomas. Immunohistochemical analysis of the KIT receptor is well established in canine mast cell tumours [39], but, to the best of the authors’ knowledge, it has not been investigated in larger case numbers of canine SCC so far. There is just one case report of a dog with an oral collision tumour of SCC and malignant melanoma that examined the expression of the KIT receptor in the neoplastic cells—but only melanocytic cells were positive [40]. In human dermal SCC, only 3 of 22 neoplasms showed expression of the KIT receptor [41]. Unfortunately, several cases of dSCC in the present study were not diagnosed in our laboratory, and samples were not available for further analyses, but prospective case collections should focus on this to achieve a better understanding of the pathogenetic correlations.

In summary, this is the first time that a prognostically relevant germline mutation has been detected for a specific canine tumour (dSCC in black giant schnauzers). However, the method of copy number analysis of KITLG does not say anything about the distribution of the chromosome sets. Thus, including CNV analysis of the KITLG locus in breeding decisions is complex, and the prevention of squamous cell carcinoma of the digit in giant schnauzers remains challenging for breeders. Nevertheless, this diagnostic test can help to assess the individual risk of developing the disease and to sensitise owners accordingly if the CN value is high. It may encourage owners to screen their dogs for neoplasms if they understand that there is a well-known breed predisposition. Thus, a regular medical check-up (adspection and palpation) of the toes is recommended especially in such high-risk dogs, and early surgery may prevent metastases.

## 5. Conclusions

In summary, detecting the copy number variation of the KITLG locus in black giant schnauzers is a promising new tool to predict the individual risk of developing squamous cell carcinoma of the digit. Further research is necessary to make comparable statements in standard schnauzers and to understand the protective mechanisms in miniature schnauzers.

## Data Availability

The raw data of the results presented in this study are available on request from the corresponding author.

## Supplementary Materials

The following supporting information can be downloaded at: https://www.mdpi.com/article/10.3390/vetsci10020147/s1, Figure S1: Scatterplot, KITLG CNV value.

## References

[1] Grassinger J.M., Floren A., Müller T., Cerezo-Echevarria A., Beitzinger C., Conrad D., Törner K., Staudacher M., Aupperle-Lellbach H. (2021). Digital Lesions in Dogs: A Statistical Breed Analysis of 2912 Cases. Vet. Sci..

[2] Wobeser B.K., Kidney B.A., Powers B.E., Withrow S.J., Mayer M.N., Spinato M.T., Allen A.L. (2007). Diagnoses and clinical outcomes associated with surgically amputated canine digits submitted to multiple veterinary diagnostic laboratories. Vet. Pathol..

[3] Henry C.J., Brewer W.G., Whitley E.M., Tyler J.W., Ogilvie G.K., Norris A., Fox L.E., Morrison W.B., Hammer A., Vail D.M. (2005). Canine digital tumors: A veterinary cooperative oncology group retrospective study of 64 dogs. J. Vet. Intern. Med..

[4] Belluco S., Brisebard E., Watrelot D., Pillet E., Marchal T., Ponce F. (2013). Digital squamous cell carcinoma in dogs: Epidemiological, histological, and immunohistochemical study. Vet. Pathol..

[5] Marconato L., Murgia D., Finotello R., Meier V., Morello E.M., Pisoni L., Foglia A., Guerra D., Chalfon C., Aralla M. (2021). Clinical Features and Outcome of 79 Dogs With Digital Squamous Cell Carcinoma Undergoing Treatment: A SIONCOV Observational Study. Front. Vet. Sci..

[6] Chiu O., Wilcock B.P., Wilcock A.E., Edwards A.M. (2022). Breed predilections and prognosis for subungual squamous cell carcinoma in dogs. Can. Vet. J..

[7] Frese K., Frank H., Eskens U. (1983). Plattenepithelkarzinome der Zehen beim Hund. Dtsch. Tierarztl. Wochenschr..

[8] Webb J.L., Burns R.E., Brown H.M., Leroy B.E., Kosarek C.E. (2009). Squamous cell carcinoma. Compend. Contin. Educ. Vet..

[9] Lecerf P., Richert B., Theunis A., André J. (2013). A retrospective study of squamous cell carcinoma of the nail unit diagnosed in a Belgian general hospital over a 15-year period. J. Am. Acad. Dermatol..

[10] O’Brien M.G., Berg J., Engler S.J. (1992). Treatment by digital amputation of subungual squamous cell carcinoma in dogs: 21 cases (1987–1988). J. Am. Vet. Med. Assoc..

[11] Nagamine E., Hirayama K., Matsuda K., Okamoto M., Ohmachi T., Uchida K., Kadosawa T., Taniyama H. (2017). Invasive Front Grading and Epithelial-Mesenchymal Transition in Canine Oral and Cutaneous Squamous Cell Carcinomas. Vet. Pathol..

[12] Jesinghaus M., Strehl J., Boxberg M., Brühl F., Wenzel A., Konukiewitz B., Schlitter A.M., Steiger K., Warth A., Schnelzer A. (2018). Introducing a novel highly prognostic grading scheme based on tumour budding and cell nest size for squamous cell carcinoma of the uterine cervix. J. Pathol. Clin. Res..

[13] Cerezo-Echevarria A., Grassinger J.M., Beitzinger C., Klopfleisch R., Aupperle-Lellbach H. (2020). Evaluating the Histologic Grade of Digital Squamous Cell Carcinomas in Dogs with Dark and Light Haircoat-A Comparative Study of the Invasive Front and Tumor Cell Budding Systems. Vet. Sci..

[14] Madewell B.R., Pool R.R., Theilen G.H., Brewer W.G. (1982). Multiple subungual squamous cell carcinomas in five dogs. J. Am. Vet. Med. Assoc..

[15] Liu S.K., Hohn R.B. (1968). Squamous cell carcinoma of the digit of the dog. J. Am. Vet. Med. Assoc..

[16] Cerezo-Echevarria A., Kehl A., Beitzinger C., Müller T., Klopfleisch R., Aupperle-Lellbach H. (2023). Evaluating the Histologic Grade of Digital Squamous Cell Carcinomas in Dogs and Copy Number Variation of KIT Ligand—A Correlation Study. Vet. Sci..

[17] Aupperle-Lellbach H., Grassinger J.M., Floren A., Törner K., Beitzinger C., Loesenbeck G., Müller T. (2022). Tumour Incidence in Dogs in Germany: A Retrospective Analysis of 109,616 Histopathological Diagnoses (2014–2019). J. Comp. Pathol..

[18] Paradis M., Scott D.W., Breton L. (1989). Squamous cell carcinoma of the nail bed in three related giant schnauzers. Vet. Rec..

[19] Corchado-Cobos R., García-Sancha N., González-Sarmiento R., Pérez-Losada J., Cañueto J. (2020). Cutaneous Squamous Cell Carcinoma: From Biology to Therapy. Int. J. Mol. Sci..

[20] Liu D., Xiong H., Ellis A.E., Northrup N.C., Dobbin K.K., Shin D.M., Zhao S. (2015). Canine spontaneous head and neck squamous cell carcinomas represent their human counterparts at the molecular level. PLoS Genet..

[21] Esteban-Villarrubia J., Soto-Castillo J.J., Pozas J., San Román-Gil M., Orejana-Martín I., Torres-Jiménez J., Carrato A., Alonso-Gordoa T., Molina-Cerrillo J. (2020). Tyrosine Kinase Receptors in Oncology. Int. J. Mol. Sci..

[22] Letard S., Yang Y., Hanssens K., Palmérini F., Leventhal P.S., Guéry S., Moussy A., Kinet J.-P., Hermine O., Dubreuil P. (2008). Gain-of-function mutations in the extracellular domain of KIT are common in canine mast cell tumors. Mol. Cancer Res..

[23] Beadling C., Jacobson-Dunlop E., Hodi F.S., Le C., Warrick A., Patterson J., Town A., Harlow A., Cruz F., Azar S. (2008). KIT gene mutations and copy number in melanoma subtypes. Clin. Cancer Res..

[24] Chetty R., Serra S. (2016). Molecular and morphological correlation in gastrointestinal stromal tumours (GISTs): An update and primer. J. Clin. Pathol..

[25] Takanosu M., Amano S., Kagawa Y. (2016). Analysis of c-KIT exon 11 mutations in canine gastrointestinal stromal tumours. Vet. J..

[26] Amagai Y., Tanaka A., Jung K., Matsuda A., Oida K., Nishikawa S., Jang H., Ishizaka S., Matsuda H. (2014). Production of stem cell factor in canine mast cell tumors. Res. Vet. Sci..

[27] Weich K., Affolter V., York D., Rebhun R., Grahn R., Kallenberg A., Bannasch D. (2020). Pigment Intensity in Dogs is Associated with a Copy Number Variant Upstream of KITLG. Genes.

[28] Gorenjak M., Fijačko N., Bogomir Marko P., Živanović M., Potočnik U. (2021). De novo mutation in KITLG gene causes a variant of Familial Progressive Hyper- and Hypo-pigmentation (FPHH). Mol. Genet. Genomic Med..

[29] Ling J., Zhang L., Chang A., Huang Y., Ren J., Zhao H., Zhuo X. (2022). Overexpression of KITLG predicts unfavorable clinical outcomes and promotes lymph node metastasis via the JAK/STAT pathway in nasopharyngeal carcinoma. Lab. Investig..

[30] Yang S., Li W., Dong F., Sun H., Wu B., Tan J., Zou W., Zhou D. (2014). KITLG is a novel target of miR-34c that is associated with the inhibition of growth and invasion in colorectal cancer cells. J. Cell. Mol. Med..

[31] Salomonsson A., Jönsson M., Isaksson S., Karlsson A., Jönsson P., Gaber A., Bendahl P.-O., Johansson L., Brunnström H., Jirström K. (2013). Histological specificity of alterations and expression of KIT and KITLG in non-small cell lung carcinoma. Genes Chromosomes Cancer.

[32] Pös O., Radvanszky J., Buglyó G., Pös Z., Rusnakova D., Nagy B., Szemes T. (2021). DNA copy number variation: Main characteristics, evolutionary significance, and pathological aspects. Biomed. J..

[33] Macé A., Kutalik Z., Valsesia A. (2018). Copy Number Variation. Methods Mol. Biol..

[34] Brancalion L., Haase B., Wade C.M. (2022). Canine coat pigmentation genetics: A review. Anim. Genet..

[35] Karyadi D.M., Karlins E., Decker B., vonHoldt B.M., Carpintero-Ramirez G., Parker H.G., Wayne R.K., Ostrander E.A. (2013). A copy number variant at the KITLG locus likely confers risk for canine squamous cell carcinoma of the digit. PLoS Genet..

[36] Conrad D., Kehl A., Beitzinger C., Metzler T., Steiger K., Pfarr N., Fischer K., Klopfleisch R., Aupperle-Lellbach H. (2022). Molecular Genetic Investigation of Digital Melanoma in Dogs. Vet. Sci..

[37] R Core Team (2022). A Language and Environment for Statistical Computing.

[38] Davison A.C., Hinkley D.V. (2000). Bootstrap Methods and Their Application.

[39] Freytag J.O., Queiroz M.R., Govoni V.M., Pereira I.V.A., Pulz L.H., de Francisco Strefezzi R., Queiroga F.L., Cogliati B. (2021). Prognostic value of immunohistochemical markers in canine cutaneous mast cell tumours: A systematic review and meta-analysis. Vet. Comp. Oncol..

[40] Rodríguez F., Castro P., Ramírez G.A. (2016). Collision Tumour of Squamous Cell Carcinoma and Malignant Melanoma in the Oral Cavity of a Dog. J. Comp. Pathol..

[41] Goto K., Ishikawa M., Hamada K., Muramatsu K., Naka M., Honma K., Sugino T. (2021). Comparison of Immunohistochemical Expression of Cytokeratin 19, c-KIT, BerEP4, GATA3, and NUTM1 Between Porocarcinoma and Squamous Cell Carcinoma. Am. J. Dermatopathol..

