# Supplementary figures and images for: KITLG Copy Number Germline Variations in Schnauzer Breeds and Their Relevance in Digital Squamous Cell Carcinoma in Black Giant Schnauzers
Source: Vet Sci. 2023 Feb 11;10(2):147. doi: 10.3390/vetsci10020147 (PMC9966798; doi:10.3390/vetsci10020147)

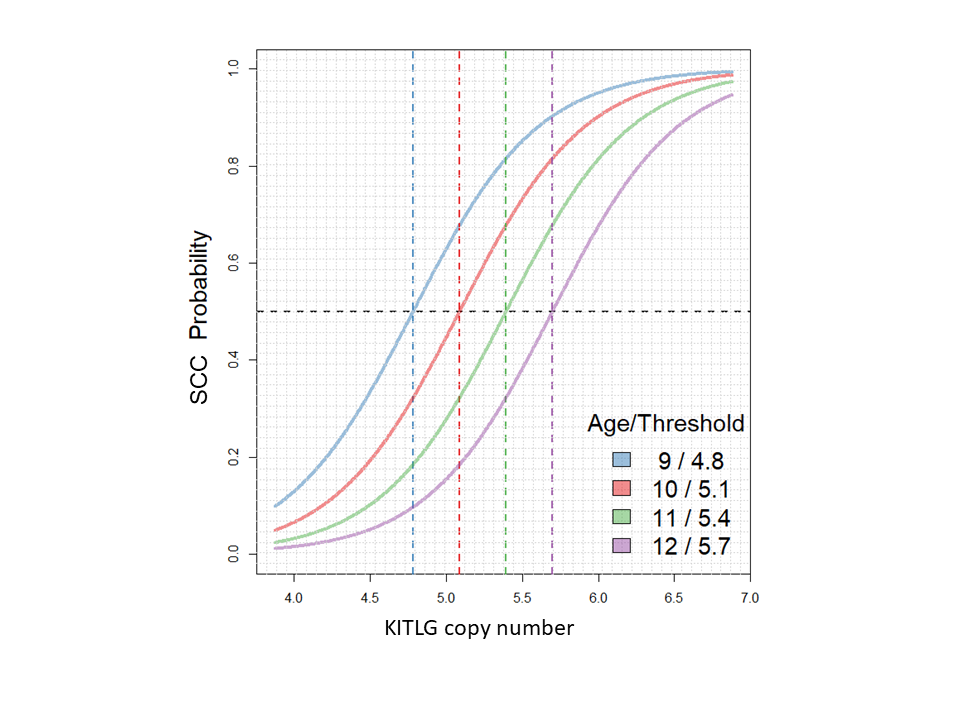

Supplement: Supplementary file 1 [file vetsci-10-00147-s001.zip › vetsci-2202698-supplementary Figure S1.TIF]
